# Comprehensive Genome-Scale Analysis of Esophageal Carcinoma With Esophageal Tissue-Resident Micro-Environment Discrepancy

**DOI:** 10.3389/fmicb.2022.859352

**Published:** 2022-05-02

**Authors:** Huiqin Yang, Xing Jin, Tao Cheng, Guangyao Shan, Chunlai Lu, Jie Gu, Cheng Zhan, Fengkai Xu, Di Ge

**Affiliations:** Department of Thoracic Surgery, Zhongshan Hospital, Fudan University, Shanghai, China

**Keywords:** esophageal squamous carcinoma, tissue-resident flora, esophageal tissue-resident micro-environment, LASSO analysis, R language software

## Abstract

To figure out the molecular mechanism in the esophageal squamous carcinoma (ESCC) with the discrepancy in the tissue-resident microbiota, we selected clinical features, RNA sequences, and transcriptomes of ESCC patients from The Cancer Genome Atlas (TCGA) website and detailed tissue-resident microbiota information from The Cancer Microbiome Atlas (*n* = 60) and explored the infiltration condition of particular microbiota in each sample. We classified the tissue-resident micro-environment of ESCC into two clusters (A and B) and built a predictive classifier model. Cluster A has a higher proportion of certain tissue-resident microbiota with comparatively better survival, while Cluster B has a lower proportion of certain tissue-resident microbiota with comparatively worse survival. We showed traits of gene and clinicopathology in the esophageal tissue-resident micro-environment (ETM) phenotypes. By comparing the two clusters’ molecular signatures, we find that the two clusters have obvious differences in gene expression and mutation, which lead to pathway expression discrepancy. Several pathways are closely related to tumorigenesis. Our results may demonstrate a synthesis of the infiltration pattern of the esophageal tissue-resident micro-environment in ESCC. We reveal the mechanism of esophageal tissue-resident microbiota discrepancy in ESCC, which may contribute to therapy progress for patients with ESCC.

## Introduction

Esophageal cancer (EC) was a commonly seen upper gastrointestinal tract malignant tumor which was responsible for an estimated 572,034 new cases (3.2% of all) and 508,558 deaths (5.3% of total cancer death) in 2018. EC ranked sixth on the cause of death from cancer worldwide (Abnet et al., 2018). Esophageal squamous carcinoma (ESCC) is different from esophageal adenocarcinoma (EADC) in geographic patterns, etiologies, and time trends, which account for nearly 90% of the cases of EC in East Asia (Bray et al., 2018). The prognosis for ESCC was poor, and there are limited options for ESCC patients who have sensitive responses to the risk of relapse after surgical removal (Park et al., 2018).

The occurrence of EC is significantly correlated with nutritional status and eating habits. The number of gastrointestinal microbiota of different types is also affected by the above factors (Hooper et al., 2001). The human body can provide an ecosystem for the survival of 10–100 trillion microorganisms (Neish, 2009). The human gastrointestinal microbiota were reported to be not only closely related to multiple local diseases of the gastrointestinal tract disease, such as inflammatory bowel disease, reflux-related esophagitis, and Barrett’s esophagus, but also some systemic diseases such as diabetes, non-alcoholic fatty liver disease (NAFLD), and some cancers (Cani et al., 2008; Henao-Mejia et al., 2012; Parkes et al., 2012; Gao et al., 2015). Comprehensively, gastrointestinal microbiota refer to the microbial environment in the human digestive tract, including the esophagus.

Increasing researchers have demonstrated that the esophageal tissue-resident microbiota have an important role in carcinogenesis and the pathophysiology of cancers. The type and proportion of esophageal tissue-resident microbiota might be of great value in predicting the genetic and prognosis of EC, which is not well explored yet. In this study, we analyzed the esophageal tissue-resident microbiota and multi-omics data of esophageal squamous cell carcinoma (SCC) from The Cancer Genome Atlas (TCGA) and The Cancer Microbiome Atlas (TCMA) databases. We attempted to explore the internal relationship and molecular mechanism between them. We hope our results can build a reliable model for predicting the survival and prognosis of patients with ESCC.

## Materials and Methods

### Proportion of Esophageal Tissue-Resident Microbiota

The esophageal tissue-resident microbiota data we used were from TCMA after comparing and integrating data from multiple next-generation sequencing (NGS) platforms and various sample types. TCMA database isolated and experimentally validated the tissue-resident component of these datasets, thus producing a public resource of computationally decontaminated microbial profiles in TCGA tissue samples. The TCMA database has various classified tissue-resident microbiota data, and esophageal microbiota information is separated from other tissues. TCMA directly provided the proportion of certain microbiota in each sample. The data special for esophageal microbiota can be found at https://tcma.pratt.duke.edu [main Streamlit (duke.edu)] (Dohlman et al., 2021). We found the TCMA database and detailed information about how the microbiota data are normalized in Method details in this paper: Dohlman et al. (2021). We removed normal tissue data and kept the tumor tissue data. We matched serial number of each sample in TCMA and TCGA, which was realized by using the R dplyr and tidyverse package.

### Expression of Esophageal Squamous Carcinoma Datasets

RNA-seq gene expression, nucleotide variation, copy number variation (CNV), miRNA expression, survival, and clinical information datasets of patients with ESCC (*n* = 60) were retrieved from TCGA data portal and UCSC Xena Browser. All data were downloaded from https://xenabrowser.net/datapages/?cohort=TCGA%20Esophageal%20Cancer%20(ESCA)&removeHub=https%3A%2F%2Fxena.treehouse.gi.ucsc.edu%3A443 or UCSC Xena^1^ or Esophageal Cancer—Patient Version—National Cancer Institute. We removed patients with insufficient or unavailable clinical and survival information. We used the R tidyverse package for data processing (Colaprico et al., 2015).

### Consensus Groups for Esophageal Tissue-Resident Micro-Environment Infiltration, Esophageal Tissue-Resident Microbiota, and Heatmaps

We performed the unsupervised consensus clustering based on esophageal tissue-resident microbiota proportion. The most appropriate k value was found to be 2 after checking out the heatmaps of the consensus matrices and the CDF plot. Heatmaps are based on hierarchical clustering of β-values using Euclidean distance and Ward’s algorithm (R package gplots). We used the proportion of ambiguously clustered pairs (PAC) to determine the optimal clustering number. The parameter of reps was set by 1,000 times in Consensus Cluster Plus. Therefore, clustering trees were bootstrapped 1,000 times to ensure the robustness of clustered nodes. The code can be found in https://www.linkedin.com/pulse/how-use-pac-measure-consensus-clustering-yasin-%C5%9Fenbabao%C4%9Flu. The pheatmap package was used to plot the five esophageal tissue-resident microbiota clustering patterns from different patients.

### Differentially Expressed Genes Analysis

Differentially expressed genes and miRNAs in different cluster groups were identified by the package limma, which implements the Benjamini and Hochberg (BH) method to compute gene expression changes with moderated *t*-test and adjust the *P*-value as a false discovery rate (FDR) (Ritchie et al., 2015). The cutoff criteria were an absolute value of log fold change (FC) > 1 but adjusted *P* < 0.05 for differential expression. We choose log (FC) > 1 is that multiple researchers had chosen log (FC) > 1 as a standard to distinguish upregulated genes, while log (FC) < -1 means downregulated genes (de Abreu Neto et al., 2017; Praz et al., 2018). The cluster Profiler package was used for analyzing the functional enrichment of the detected DEGs. The cutoff for Gene Ontology (GO) and Kyoto Encyclopedia of Genes and Genomes (KEGG) terms was adjusted *P*-value < 0.05 and FDR < 0.05. We have improved our data processing with the combat algorithm in the SVA package in R to wipe out batch effects. To decrease the FDR, Benjamini–Hochberg procedure, based on 10,000 permutations, was used to calculate and adjust the enrichment *p*-values for multiple testing.

### Differentially Mutated Genes Analysis

The simple nucleotide variation data were stratified into two groups, and their mutational patterns were investigated separately. Differentially mutated genes were detected using Fisher’s exact test and visualized by the maftools package. Besides, mutational load and CNV were calculated for every patient, and the differences between the two groups were explored.

### Construction of the Predictive Model

After taking the intersection of the DEGs and 137 genes, we obtained 10 DEGs (IF-DEGs). Based on the DEGs, we further performed gene selection using the least absolute shrinkage and selection operator (LASSO) with fivefold cross-validation linear regression (Tibshirani, 1996; Vasquez et al., 2016) implemented in R glmnet package. This method uses an L1 penalty to shrink the regression coefficients for unimportant genes to zero. This results in a parsimonious model where only the genes with the strongst associations with the outcome will be selected. Genes were selected from the LASSO linear regression models with the optimal value of lambda from leave-one-out cross-validation at each taxonomic level. Covariates included in the traditional linear regression model were controlled in the LASSO taxa selection process. Subsequent binary logistic regression analysis is also performed using a *p*-value of 0.05 as a threshold for statistical significance. Model selection was repeated using a stepwise forward and backward approach to assess whether the variables included in the final model were influenced by the approach for the multivariate analysis (sensitivity analysis). The models were compared using a receiver operating characteristic curve (ROC) analysis to determine the most predictive model. The model is used to predict whether ESCC patients mutualize specific esophageal tissue-resident micro-environment.

### Data Processing

All statistical analyses **were conducted in R software (version 4.0.3). The comparison of the clinical features of the ESCC** patients between the two clusters was made, of which categorical variables were compared by Chi-square test or Fisher**’s exact test when Student’s *t***-test compared appropriate and continuous variables. S**tudent’s *t***-test was also used to compare continuous variables such as the mutational load, and l**og-rank test was used to compare overall survival between the two groups in the Kaplan–Meier survival analysis. All** **the tests used in our study were two-sided, and the significance threshold of the *p***-value was set as 0.05.

## Results

### the Landscape of the Esophageal Tissue-Resident Microbiota Clustering in Esophageal Squamous Carcinoma

**FIGURE 1:**
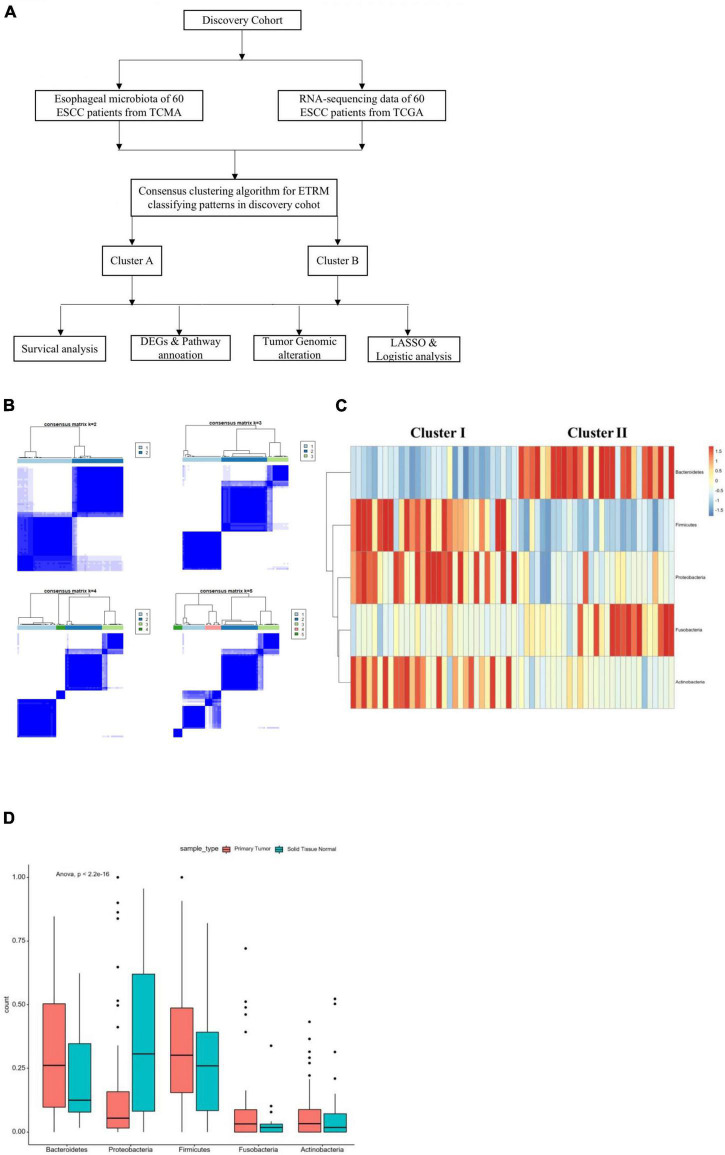
**(A)** Construction of the study. **(B)** Consensus matrixes of all sample cohorts for each k (k from 2 to 5) demonstrate the stability of clustering through 2000 HYPERLINK “javascript” hierarchical clustering. **(C)** All-randomized clustering of esophageal-tissue resident microbiota for 60 samples from both TCGA and TCMA. **(D)** Proportion of the most significant esophageal tissue-resident microbiota in the primary tumor and solid normal tissue according to the best clustering (Table 1). Basic characteristics of the ESCC patients in the two infiltration groups from the TCGA database.

**FIGURE 2:**
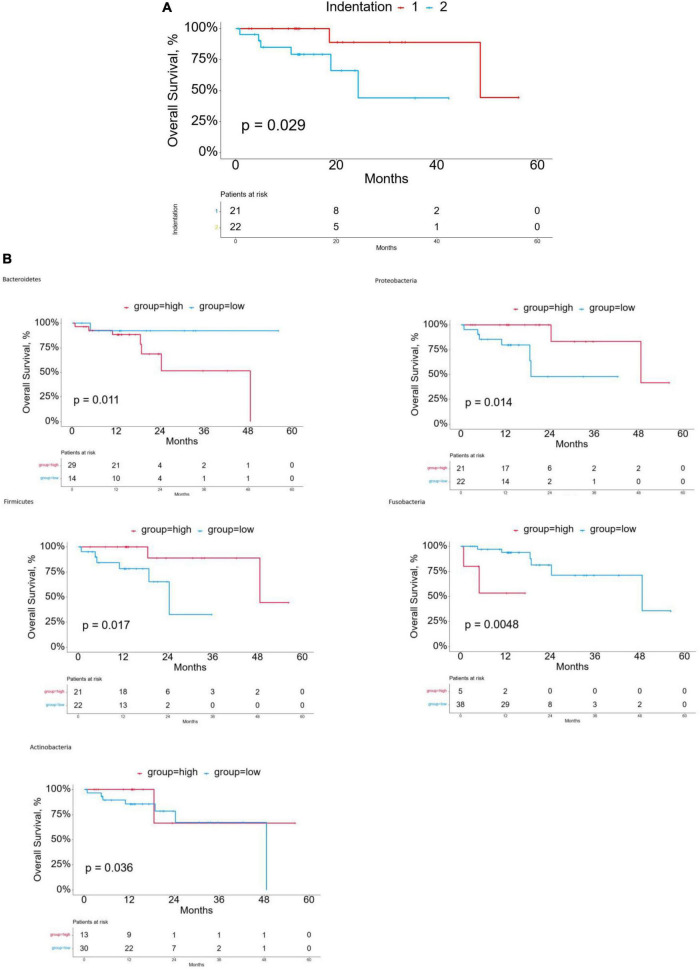
**(A)** Kaplan-Meier curves for overall survival (OS) of cluster groups in the discovery cohort. **(B)** Kaplan-Meier curves for overall survival (OS) based on esophageal tissue-resident microbiota.

The design of this study is shown in Figure 1A. We matched 60 patients from TCMA with TCGA. We used the proportion of esophageal tissue-resident microbiota to show each sample’s relevant abundance by unsupervised consensus clustering, which can systematically show the characteristic of each ESCC patient’s esophageal tissue-resident microbiota clustering phenotype. We conducted the clustering procedure by the proportion of the esophageal tissue-resident microbiota subsets with PAC to choose the optimal and stable cluster number (Figure 1B). The cluster of k-means ensured we gained the optimal sequestration by dichotomizing the training cohort, separating the 60 patients into two groups called cluster I (*n* = 31) and cluster II (*n* = 29). The result also showed the most enriched five kinds of esophageal tissue-resident microbiota, namely, Bacteroidetes, Proteobacteria, Firmicutes, Fusobacteria, and Actinobacteria. The samples were divided into two clusters according to the distribution of these five kinds of esophageal tissue-resident microbiota in each sample. Cluster I had a high proportion of Proteobacteria, Firmicutes, and Actinobacteria and a low proportion of Bacteroidetes and Fusobacteria, while cluster II had a low proportion of Proteobacteria, Firmicutes, and Actinobacteria and a high proportion of Bacteroidetes and Fusobacteria (Figure 1C). We found out that Bacteroidetes and Fusobacteria belong to Bacillus, while Proteobacteria, Firmicutes, and Actinobacteria are not. Cluster I and Cluster II can also be entitled to the low Bacillus proportion cluster and the high Bacillus proportion cluster. We also compared the esophageal tissue-resident microbiota in the patients with primary tumor or solid normal tissue (Figure 1D). There is a significant difference between the two cluster groups in OS (*p* = 0.029; Figure 2A). The “hot area,” cluster I, was infiltrated with a high proportion of the three kinds of esophageal tissue-resident microbiota, so we selected the color red to show the activated distribution in these esophageal tissue-resident microbiota. In contrast, the “cold area” had a relatively tenuous proportion of the three kinds of esophageal tissue-resident microbiota. Thus, we used the color blue to designate tenuous esophageal tissue-resident microbiota proportion. We drew the Kaplan–Meier curve under the circumstances of the esophageal tissue-resident microbiota subsets. The results demonstrated that patients with a high proportion of Proteobacteria, Firmicutes, and Actinobacteria had better OS (*p* = 0.014, 0.017, and 0.036). In comparison, patients with a high proportion of Bacteroidetes and Fusobacteria had worse OS (*p* = 0.011 and 0.0048) (Figure 2B).

**TABLE 1:** Clinical features of the two clusters.

	1	2	p.overall
	*N* = *21*	*N* = *22*	
sex:			0.457
female	5 (23.8%)	3 (13.6%)	
male	16 (76.2%)	19 (86.4%)	
age	63.7 (10.3)	64.0 (13.1)	0.937
age_median:			1.000
older	12 (57.1%)	13 (59.1%)	
younger	9 (42.9%)	9 (40.9%)	
BMI	25.2 (21.8;32.5)	22.1 (20.2;24.2)	0.023
smoke:			0.826
No	15 (71.4%)	14 (63.6%)	
Yes	6 (28.6%)	8 (36.4%)	
T:			0.500
T1	6 (28.6%)	2 (9.09%)	
T2	3 (14.3%)	5 (22.7%)	
T3	11 (52.4%)	13 (59.1%)	
T4	1 (4.76%)	2 (9.1%)	
N:			0.648
N0	12 (57.1%)	11 (50.0%)	
N1	5 (23.8%)	9 (40.9%)	
N2	2 (9.52%)	1 (4.55%)	
N3	2 (9.52%)	1 (4.55%)	
M:			0.222
M0	15 (71.4%)	18 (81.8%)	
M1	0 (0.00%)	1 (4.55%)	
M1a	3 (14.3%)	1 (4.55%)	
MX	3 (14.3%)	2 (9.1%)	
stage2:			0.392
I	5 (25.0%)	2 (9.52%)	
II	9 (45.0%)	11 (52.4%)	
III	6 (30.0%)	6 (28.6%)	
IV	0 (0.00%)	2 (9.52%)	
OS:			0.240
0	19 (90.5%)	16 (72.7%)	
1	2 (9.52%)	6 (27.3%)	
OS.time	13.4 (12.8;23.5)	12.7 (5.12;18.5)	0.123

### Clinical Features of the Esophageal Tissue-Resident Microbiota Clustering in Esophageal Squamous Carcinoma

Concerning baseline traits, we studied the age, sex, BMI, smoking or not, TNM stages, and stage distribution of the clustering groups (Table 1). The median data of the diagnosis age were 63.7 years in cluster I, but 64 years in cluster II (Chi-square, *p* = 0.837). The median data of the BMI were 25.2 in cluster I, but 22.1 in cluster II (*p* = 0.023). There was no significant difference in staging (Fisher’s, *p* = 0.392) or TNM stage (Fisher’s, *p* = 0.5, 0.648, and 0.222) between the two groups, nor in sex or whether smoking (*p* = 0.457 and 0.826), which showed that the distribution in the cluster groups is independent of age, sex, smoking, TNM stage, and stage, but the distribution in the cluster groups may be related to BMI. In order to remove the effect of the cofounder BMI on the microbiota of the identified two clusters, we performed multivariate Cox regression analysis. The result showed that the OS does not correspond to the BMI [HR ratio and 95% CI: 1(0.94–1.07), *P* = 0.945].

### Differentially Expressed Genes and Functional Annotation

**FIGURE 3:**
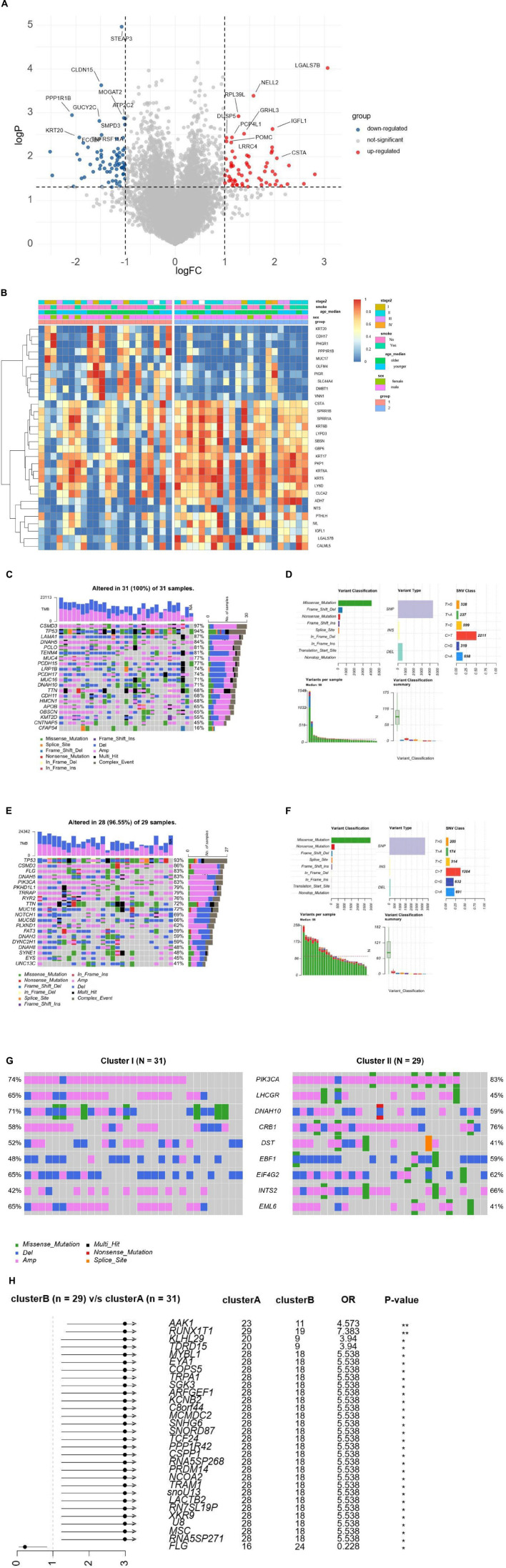
**(A)** The volcano plot demonstrates the DEGs of clusters A and B. **(B)** Unsupervised clustering of 44 ESCC patients from TCGA and TMCA. Clinical-pathologic characteristics contain age, stage, smoking, and cluster groups. **(C)** The waterfall plots demonstrate the proportion of genomic mutations and certain types of mutations in cluster I. **(D)** Detailed somatic genomic mutations and variations of copy number analysis of cluster I. **(E)** The waterfall plots demonstrating the proportion of genomic mutations and certain types of mutations in cluster II. **(F)** Detailed somatic genomic mutations and variations of copy number analysis of cluster II. **(G)** The waterfall plots demonstrate the genomic alterations including somatic genomic mutations and variations of copy number in the two cluster cohorts. **(H)** The forest plot shows the value of each co-related genome of prognosis by corresponding hazard ratio (HR) and Odds ratio with 95% CI.

**FIGURE 4:**
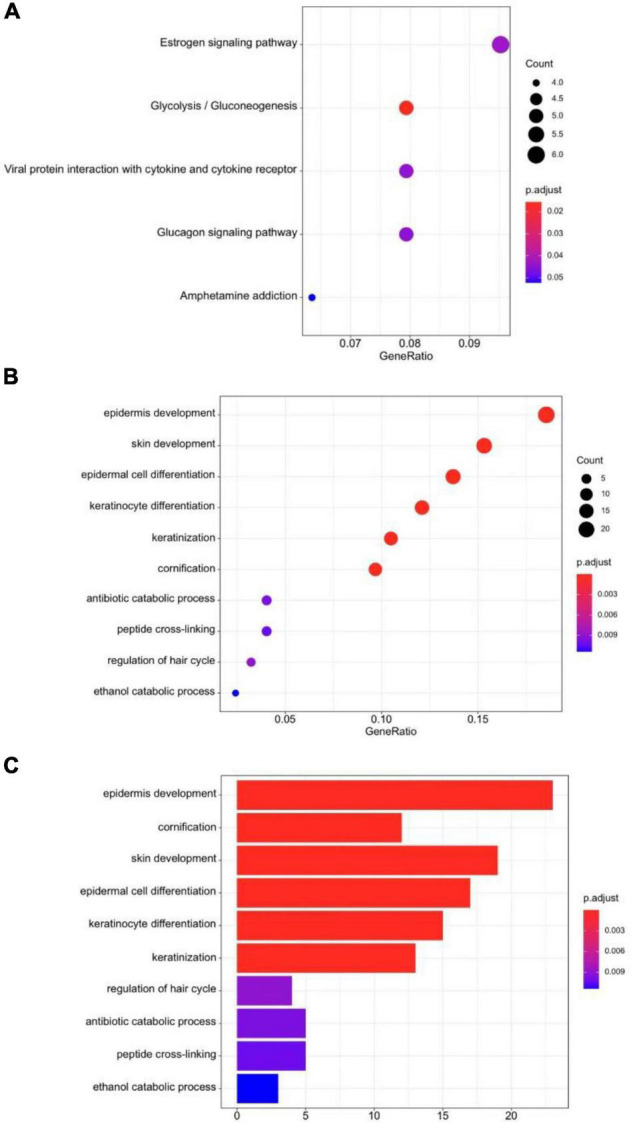
**(A)** KEGG dot-plot of functional enrichment in co-related pathways analysis of DEGs. **(B)** GO dot-plot of functional enrichment in co-related pathways analysis of DEGs. **(C)** GO bar-plot of functional enrichment in co-related pathways analysis of DEGs.

We analyzed the expression profiles for DEGs to describe the biological characteristics in two cluster groups. In total, 63 genes such as NELL2, PRL39L, GRHL3, and IGFL1 were upregulated (all adjusted *p* < 0.01) and 70 were downregulated in cluster II, including CLDN15, MOGAT2, and SMPD3 (Figure 3A). The pheatmap also demonstrated that the two clusters had a distinct expression of DEGs considering sex, age, and stages (Figure 3B). We used the R cluster profile package to analyze the enrichment of GO and KEGG in the 133 DEGs and the pathways related to tumorigenesis and intraepithelial neoplasia in cluster II. We found that epithelial cellular biological behavior correlated with tumorigenesis and intraepithelial neoplasia corresponded to the discrepant proportion of esophageal tissue-resident microbiota in cluster II (Figures 4A–C). In cluster I, several classic metabolic pathways ranked top.

### Tumor Genomic Alterations Associated With Cluster Groups

Previously reported findings showed that the number of somatic genomic mutations may be related to the response to tumorigenesis and intraepithelial neoplasia. We then explored the distribution patterns of somatic mutations and the variety of copy numbers in the two cluster groups. The median of somatic variants per sample was 98 and calculated in clusters I and II, respectively. The most mutated genes in cluster I were CSMD3, TP53, LAMA1, DNAH5, PCLO, and TENM4 (97, 94, 87, 84, 81, and 81%, respectively; Figure 3C). Missense mutation overwhelmed the majority in the variant classification. Variant type mainly was single nucleotide polymorphism, and the SNV class was C changing to T (Figures 3D,F). The most mutated genes in cluster II were TP53, CSMD3, FLG, DNAAH5, and PIK3CA (96, 86, 83, 83, and 83%, respectively; Figure 3E). Variant classification, variant type, and SNV class were similar to cluster I. In ESCC, TP53 is the essential tumoral driver gene. However, TP53 was slightly higher in cluster I with no meaningful difference in mutational frequencies of the cluster groups. The most significant DMGs were compared and listed (Figure 3G). The result showed nine DMGs such as PIK3CA (74% in cluster I, 83% in cluster II), LHCGR (65% in cluster I, 65% in cluster II), and DNAH10 (71% in cluster I, 59% in cluster II). Cluster I had more DMGs than cluster II. Only FLG was listed (Figure 3H). Based on the data, we can describe the scenario of cluster groups more comprehensively and reveal the complicated relationship between individual somatic mutation and the impact of discrepant esophageal tissue-resident microbiota on the tumor.

### Prognostic Value of Cluster Groups

**FIGURE 5:**
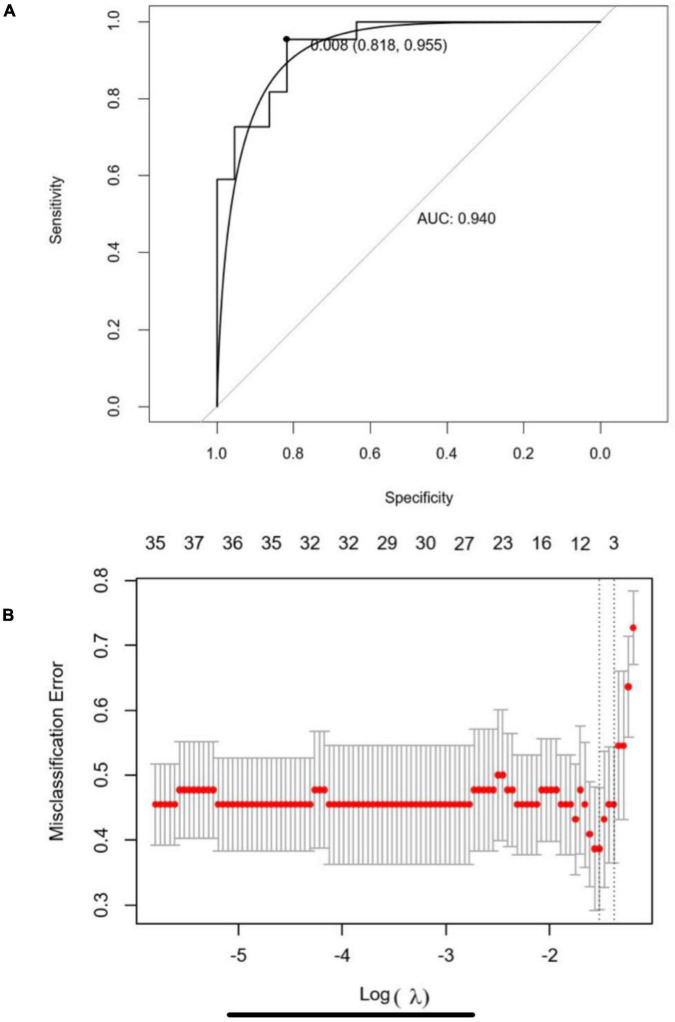
**(A)** Five-fold cross-validation for tuning parameter (λ) selection in the LASSO regression model. The partial likelihood deviance is plotted in log(λ), in which vertical lines are shown at the optimal values by minimum criteria and 1-SE criteria. **(B)** Receiver operating characteristic curve (ROC) of the LASSO-binary logistic regression model.

The optimal parameter λ of LASSO analysis was set as the smallest partial likelihood of deviance (Figure 5A). A panel of 10 DEGs including SNX3, AKIRIN2, TMEM87B, STEAP3, PPME1, LGALS7B, ARFRP1, STX11, RP11-295P9.3, and RP11-434D12.1 were employed. Subsequently, we conducted the binary logistic regression analysis to get the coefficients of each item. The predictive model could be demonstrated as the following formula: score = (0.1566 × expression level of SNX3) + (0.0265 × expression level of AKIRIN2) + (−0.0127 × expression level of TMEM87B) + (−0.329 × expression level of STEAP3) + (0.0315 × expression level of PPME1) + (0.0363 × expression level of LGALS7B) + (−0.0721 × expression level of ARFRP1) + (0.0133 × expression level of STX11) + (−0.0878 × expression level of RP11-295P9.3) + (−0.1081 × expression level of RP11-434D12.1). ROC demonstrated that the cutoff value of this model was 0.008, and the area under the curve (AUC) was 0.940 (Figure 5B). When the score is less than 0.008, the ESCC patient is more likely to have a high proportion of the three esophageal tissue-resident microbiota, classified into cluster I. If the score is higher than 0.008, the patient is more likely to have a low proportion of the three esophageal tissue-resident microbiota, classified into cluster II. The sensitivity and specificity of this model were 0.818 and 0.955, respectively.

## Discussion

Gastrointestinal microbiota are reported to have a significant impact on tumorigenesis. One research reveals that active estrogen is thought to regulate endogenous estrogen metabolism through enterohepatic circulation by the enzymatic activity of bacterial β-glucosidase, thereby affecting circulating and excreted estrogen levels which is a critical risk of breast cancer (Dabek et al., 2008). Ling et al. showed that the microbiota composition (beta diversity) remained distinctive. A few bacteria were different in abundance among the patients compared with controls despite completion of chemotherapy and presumed restoration of normal health (Chua et al., 2020). These persistent microbiota changes may have a role in the long-term well-being of childhood cancer survivors. However, the impact of these changes on subsequent health perturbations in these survivors remains unexplored (Chua et al., 2020). Bernd et al. revealed that the gastrointestinal microbiome contributes to the onset and progression of alcoholic liver disease and NAFLD and mediates complications in end-stage liver disease (Schnabl and Brenner, 2014). There appears to be an association between gastrointestinal dysbiosis and liver disease in patients. Changes in the gastrointestinal microbiome were found to cause liver disease mostly in animal models. Few have been associated with the metabolic and immunologic features of patients with NAFLD and NASH (non-alcoholic steatohepatitis) (Schnabl and Brenner, 2014). In addition, Pushalkar et al. (2018) detected specific gastrointestinal and tumor microbiome in murine models of pancreatic ductal adenocarcinoma, suggesting potential bacterial translocation from the digestive tract into the peritumoral milieu.

Each person can have 500–1000 unique microbial species representing the digestive micro-environment of individuals. Therefore, genetic analysis can measure the type and number of gastrointestinal microbiota specific to an individual. The disturbance of the gastrointestinal microbiota ecosystem can affect human health. The difference in the expression level of specific bacteria may be closely related to the occurrence of related tumors. Analyzing individual gene differences can predict the type and proportion of gastrointestinal microbiota. The incidence and prognosis of EC can be predicted by analyzing the proportion of gastrointestinal microbiota. Increasing researchers have demonstrated that gastrointestinal microbiota have an important role in carcinogenesis and the pathophysiology of extraintestinal cancers. Yu et al. (2017) found significant associations with several species, including Parvimonas micra and Solobacterium. They identified 20 microbial genetic markers used to distinguish colorectal cancer from controls and validated four markers in the Danish cohort. These four genes distinguished the CRC metagenome from the control group in the French and Austrian cohorts, whose regions under the subject operation curve (AUC) were 0.72 and 0.77, respectively. QPCR measurements of these two genes accurately identified CRC patients in an independent Chinese cohort, with AUC = 0.84 and OR = 23. These genes are enriched in the microbiome of early (I–II) patients, highlighting the potential of using fecal metagenomic biomarkers for the early diagnosis of CRC (Yu et al., 2017).

Recent researchers have found that oral and esophagus microbiota may contribute to the occurrence of ESCC (Yu et al., 2017; Ren et al., 2019). However, few findings focused on esophageal tissue-resident microbiota and the distribution in ESCC patients. This study is the first report to reveal the different esophageal tissue-resident microbiota proportions and selected the most significant kinds of esophageal tissue-resident microbiota. In order to explore and identify the molecular mechanism in the ESCC micro-bio-environment and help propel cancer prediction and inspection of ESCC, we assorted ESCC patients into different cluster groups according to the proportion of esophageal tissue-resident microbiota, trying to put the genetic mutation, expression, and other differences into the in-depth analysis in this manuscript. We demonstrate the cluster group as an independent prognostic factor for ESCC patients. In our research, the prominent OS disparity might relate to the different distribution of esophageal tissue-resident microbiota between cluster groups. The overgrowth of various kinds of bacteria is responsible for many cancers. According to Robertson et al. (2017) and Magne et al. (2020) Firmicutes and Bacteroidetes were primarily discovered in the gastrointestinal microbiota. The classification scheme Firmicutes/Bacteroidetes was used (Robertson et al., 2017; Magne et al., 2020). The Firmicutes are one of the largest bacterial phyla with a thick peptidoglycan layer. A single cytoplasmic membrane, covered by teichoic or lipoteichoic acids, makes up monoderm envelope (Megrian et al., 2020). Bacteroidetes primarily target steroids, polysaccharides, and bile acids, which aid in polysaccharide absorption and protein synthesis; it has also been proven to boost regulatory T-cell growth and defend against inflammatory reactions (Telesford et al., 2015). Obese animals and humans have a larger ratio of Firmicutes/Bacteroidetes in their gastrointestinal microbiota than normal-weight people according to a recent study, and this ratio has been recommended as an ultimate biomarker (Magne et al., 2020). On the contrary, a low Firmicutes/Bacteroidetes ratio is commonly seen in some severe inflammation diseases. A low-level inflammation reaction can be observed with a high Firmicutes/Bacteroidetes ratio (Arneth et al., 2019). Proteobacteria is Gram-negative, indicating the presence of lipopolysaccharide in the outer membrane (Rizzatti et al., 2017). Research has revealed that low-grade inflammation is sustained by lipopolysaccharides (LPS). However, LSP is one of the strongest TNF-α stimulators found in the outer membrane of Gram-negative bacteria (Zhou et al., 2018). Actinobacteria is also critical in the development of the immune system. The main roles of commensal bacteria include the activation of intraepithelial lymphocytes, the generation of mucosal immunoglobulins, and promotion of a tolerogenic immune response (Binda et al., 2018). Also, Actinobacteria can modulate immune-inflammatory and autoimmune responses by inducing regulatory T cells (O’Mahony et al., 2008). In animal models, Actinobacteria in these mice stimulated the production of TNF-α in lipopolysaccharide-stimulated macrophages, promoting an oxidative burst that enhanced the phagocytosis of peritoneal macrophages (Cano et al., 2013). In the present research, we expect that the high concentration of TNF-α may contribute to the immune defense in inhibiting tumorigenesis. Several metagenomic sequencing studies have shown that increased Fusobacteria abundance is positively associated with CRC mortality (Kelly et al., 2018). For example, one study found that CRC patients with high clostridium levels had significantly lower overall survival than patients with average Fusobacteria levels (*P* = 0.008) (Flanagan et al., 2014). Others found that the abundance of Fusobacteria in CRC was significantly higher than that in normal tissues adjacent to the tumor histology. This difference was also evident in stool samples. Fusobacteria enrichment was also observed in colorectal adenomas (McCoy et al., 2013; Flanagan et al., 2014). In our result, Fusobacteria had higher distribution in cluster II with worse OS. So, the proportion of Fusobacteria may evolve into a predictive detection index in ESCC patients to estimate the possible survival and prognosis.

After finding the different esophageal tissue-resident microbiota classifications in the two cluster groups, we wanted to explore the molecular mechanism, so we analyzed DEGs and related pathways. We found that the energy metabolism pathway, epidermis development pathway, and keratinization pathways closely related to tumorigenesis and intraepithelial neoplasia were enriched in cluster II. SCC can arise from more than one epidermal population, including follicle enlargement squamous cells, because overexpression of overactive KRAS mutants in different epidermal lineages induces tumors with comparable efficiency (Rognoni and Watt, 2018). The change in esophageal tissue-resident microbiota distribution may lead to enrichment in tumorigenesis pathways. Changes in tumor-associated microbiota can lead to enrichment in C/N and Toll-like receptor (TLR) signaling, which plays a critical role in gastrointestinal tumor development. Selective activation occurs in tumor tissues due to increased TLR expression in tumor cells and changes in tumor-associated microbiota stratification. Changes in the composition of the symbiotic microbiome are associated with CRC development, and epithelial calcineurin regulates the development of gastrointestinal tumors by controlling the response of the epithelium changes in microbial composition and stratification (Gang et al., 2019). The increased expression of several kinds of functional pathways in cluster II may have a connection with the disorder in the esophageal tissue-resident microbiota proportion.

It is noteworthy that cluster II had an increased estrogen pathway which may be concerned with a rise in PIK3CA mutation. Patients with hormone receptor (HR)-positive, human epidermal growth factor receptor 2 (HER2)-negative breast cancer have PIK3CA mutations in about 40% of cases. Alpelisib, a PI3Kα inhibitor, has shown anticancer efficacy in preliminary investigations (André et al., 2019). Research has found that bacteria may have a direct role in the occurrence of oncogenic mutations. Pleguezuelos-Manzano et al. (2020) revealed that *E. coli* carrying PKS virulence islands could produce colibactin’s genetic toxin. Colibactin, produced by gastrointestinal microbes, was found to cause genetic damage (deficiency in single-base substitution and insertion) to human gastrointestinal stem cells (organoid) *in vitro* when injected into them, which proved that metabolite created by gastrointestinal microorganisms may directly cause genetic mutation, causing cancer (Pleguezuelos-Manzano et al., 2020).

Our research has several limitations. First, the samples of our findings are from TCGA and TCMA databases, and the number is 60, which is not a very large sample size. More samples are required for extensive RNA-sequence and bioinformatics analysis. Second, the mechanism of how esophageal tissue-resident microbiota may impact ESCC occurrence remains uncertain. Third, we cannot investigate the gene alteration in each gastrointestinal microbiota type since bulk RNA-Seq only reflects the average expression of whole cells in the sample. Fourth, the BMI between two clusters shows a statistical difference, and we did not remove the effect of the cofounder BMI on the microbiota of the identified two clusters.

In conclusion, our research finds out that the use of esophageal tissue-resident microbiota discrepancy may differentiate the potential survival of patients with ESCC. The genomic differences between two clusters also show significant differences which lead to the discrepancy in expressions of the downstream pathways. Our findings may serve as the exploratory research on how abnormal micro-bio-environment may cause tumor formation, thus providing new clinical treatment or diagnostic standards for ESCC patients.

## Data Availability Statement

The datasets presented in this study can be found in online repositories. The names of the repository/repositories and accession number(s) can be found in the article/Supplementary Material.

## Ethics Statement

Ethics approval was waived by the Zhongshan Hospital affiliated to Fudan University.

## Author Contributions

DG and FX conceptualized the study and supervised the study. HY was involved in the data curation, investigation, and writing the manuscript. XJ was involved in the data curation, investigation, and software. TC, GS, CL, and JG were involved in the formal analysis and methodology. All authors were involved in writing the manuscript and had final approval of the submitted and published versions.

## Conflict of Interest

The authors declare that the research was conducted in the absence of any commercial or financial relationships that could be construed as a potential conflict of interest.

## Publisher’s Note

All claims expressed in this article are solely those of the authors and do not necessarily represent those of their affiliated organizations, or those of the publisher, the editors and the reviewers. Any product that may be evaluated in this article, or claim that may be made by its manufacturer, is not guaranteed or endorsed by the publisher.

## Footnotes

1
xenabrowser.net
